# Pancreaticopericardial Fistula: A Case Report and Literature Review

**DOI:** 10.1155/2016/7169341

**Published:** 2016-04-17

**Authors:** Muhammad S. Khan, Najmi Shahbaz, Hassaan A. Zia, Muhammad Hamza, Henna Iqbal, Ahmed Awab

**Affiliations:** ^1^Section of Pulmonary & Critical Care Medicine, University of Oklahoma Health Sciences Center, No. WP1310, 920 Stanton L Young Boulevard, Oklahoma City, OK 73117, USA; ^2^Dow University of Health Sciences, Mission Road, Karachi 74200, Pakistan; ^3^Department of Internal Medicine, University of Oklahoma Health Sciences Center, No. WP1310, 920 Stanton L Young Boulevard, Oklahoma City, OK 73117, USA

## Abstract

*Purpose*. Pancreaticopericardial fistula (PPF) is an extremely rare complication of acute or chronic pancreatitis. This paper presents a rare case of PPF and provides systematic review of existing cases from 1970 to 2014.* Methods*. A PubMed search using key words was performed for all the cases of PPF from January 1970 to December 2014. Fourteen cases were included in the study. The cases were reviewed for demographic characteristics, diagnostic modalities, and treatment. Descriptive analysis of these variables was performed.* Results*. Median age was 43 years. 78% were known alcoholics and 73.3% had chronic pancreatitis. Dyspnea was present in 78%. Cardiac tamponade was present in 53%; 75% of patients had known chronic pancreatitis (RR = 0.74). Surgery was associated with best treatment outcomes and 50% of patients who underwent endoscopic treatment survived.* Conclusion*. PPF is a rare disease. This paper indicates that acute cardiac tamponade in patients with history of alcoholism and chronic pancreatitis could be a sign of an existing pancreaticopericardial fistula and early surgical intervention could be life-saving.

## 1. Introduction

Pancreatic pseudocyst is a known complication of pancreatitis. Persistent leakage of pancreatic secretions can result in development of internal fistula due to spontaneous erosion into neighboring hollow viscus and cavities. Leakage from pancreatic secretions can cause significant morbidity due to malnutrition and infections. A fistula from pancreatic pseudocyst to pericardium is a rare complication and can present with cardiac tamponade. In this paper we describe a case of pancreaticopericardial fistula and review similar cases from 1970 to 2014.

## 2. Case

**Figure 1:**
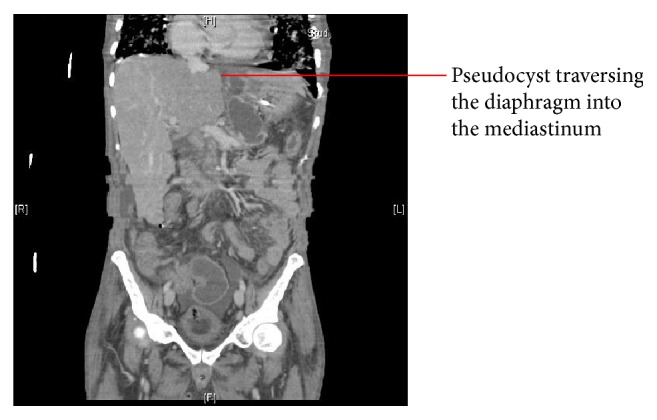
Abdominal CT showing cyst extension into mediastinum.

A middle-aged African American male presented to hospital with complaints of shortness of breath and feeling of dizziness for 3 days. Patient had known history of alcoholism and was admitted at a rehabilitation center 3 weeks before presentation for an elective detoxification. His initial course at rehabilitation center was uneventful; however, 3 days before presentation he started feeling dizzy and had gradual onset dyspnea on exertion that progressed to resting dyspnea. This was associated with sharp 3/10 epigastric pain radiating to his chest and back, which was aggravated by deep breaths. His past medical history was also significant for hypertension and episodes of delirium tremens. On admission his temperature was 37 degrees Celsius and blood pressure (BP) was 96/48 mmHg after 2-liter fluid bolus in emergency department, with pulse of 110/min, respiratory rate of 20/min, and oxygen saturation of 96% on 2 L nasal cannula. Examination was significant for decreased breath sounds in lung bases, increased vocal fremitus in left lower lobe, and dullness to percussion at lung bases bilaterally. Jugular venous distension of 7 cm when sitting and pulsus paradoxus of 15 mmHg were also noted. His white blood cell (WBC) count was 18.5 K/mm^3^ (normal range 4–11 K/cc^3^) and hemoglobin (Hb) was 10.5 g/dL (normal range Hb 13–18 g/dL), with normal electrolytes, liver function tests, cardiac enzymes, and amylase. Due to concern for cardiac tamponade, urgent transthoracic echocardiography was performed that demonstrated a large pericardial effusion with early tamponade physiology. Computed tomography (CT) scan of chest done on admission showed large pericardial effusion and reticular ground glass opacities within the lungs bilaterally. The patient was started on broad spectrum antibiotics and underwent urgent pericardiocentesis and pericardial drain placement, with 280 mL of serosanguineous fluid obtained. The fluid analysis showed 59500 red blood cells, 3750 white blood cells, lipase of 196 IU, and amylase of 101 IU. Pericardial fluid cultures and blood cultures remained negative. On day 2 of admission the pericardial drainage decreased, and the drain was removed. However, after removal the patient acutely became short of breath with oxygen saturation in low 80s despite a 15 L nonrebreather mask. He was moved to the medical intensive care unit and intubated. Repeat transthoracic echocardiography showed reaccumulation of pericardial effusion. Due to concern for pulmonary embolism, a CT angiogram of the chest was done, which was negative for pulmonary embolism but showed an abdominal pseudocyst extending from the esophageal hiatus into the mediastinum. This was subsequently followed by a CT of the abdomen, showing necrotizing pancreatitis with multiloculated pseudocyst formation. The largest fluid collection was 4.1 cm × 3.6 cm traversing the mediastinal plane concerning for pancreaticopleural-pericardial fistula (Figure 1).

**Figure 2:**
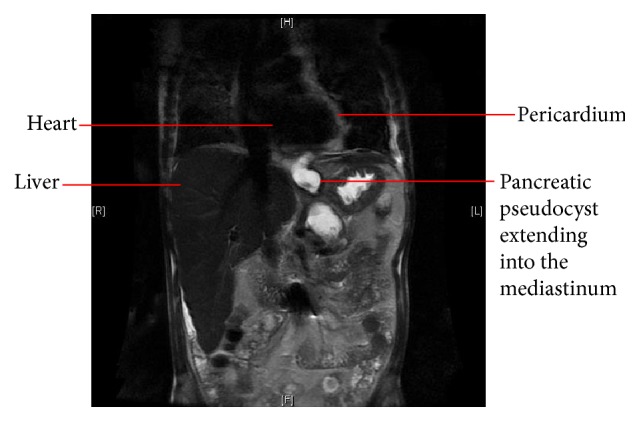
MRCP showing cyst extension into the mediastinum.

To further elaborate the pancreatic cyst and fistula, magnetic resonance cholangiopancreatography (MRCP) was also done which confirmed the findings of a large pseudocyst extending into the mediastinum (Figure 2).

The patient was subsequently extubated and moved to the medicine floor. Endoscopic retrograde cholangiopancreatography (ERCP) was performed to visualize the fistulous tract but was limited due to presence of a calculus in ventral pancreatic duct. Endoscopic ultrasound for cystgastrostomy was attempted and showed a fistulous tract towards the left pleural space. A 4 cm 7 French double-pigtail Zimmon stent was successfully placed into the pseudocyst cavity for decompression. The patient's symptoms improved after this procedure and he was discharged from the hospital on day 14. Unfortunately the patient was lost to follow-up in our clinic.

## 3. Materials and Methods

An extensive PubMed search was performed for all the cases of pancreaticopericardial fistula (PPF) using the key words “pancreatic fistula”, “pericardial tamponade”, and “chronic pancreatitis” from January 1970 to December 2014. A total of 15 cases were found. Fourteen cases were included in the study; one case was excluded as the pericardial effusion was not from fistulous tract. The cases were reviewed for age; sex; alcohol abuse; past history of chronic pancreatitis; acute pancreatitis on admission; presenting symptoms; associated symptoms of fever, abdominal pain, and chest pain; and hypotension, serum amylase levels, tamponade physiology on echocardiography, pericardiocentesis performed, pericardial amylase levels, diagnostic modality used for confirming the fistula, type of treatment offered, and patient outcomes of recovery versus death. Missing variables were marked as “unknown.” Finally a descriptive analysis of the variables was done.

## 4. Results

A total of 15 cases of PPF were found from 1971 to 2014 including our case (Table 1). All reported cases were of male gender. Median age was found to be 43 years. Seventy-eight percent of patients had history of alcoholism; 73.3% cases had a known diagnosis of chronic pancreatitis. Dyspnea was the chief presenting symptom (78%) followed by hypotension (53%), chest pain (50%), fever (43%), abdominal pain (26.6%), syncope (6.6%), weight loss (6.6%), and ascites (6.6%). Acute pancreatitis was present in 42% of cases. Cardiac tamponade was present in a total of eight cases, was negative in five cases, and was unknown in two cases. Of the patients presenting with cardiac tamponade, 75% had a past medical history of chronic pancreatitis (relative risk = 0.74, 95% confidence interval: 0.32 to 1.67, and *p* value = 0.46). Of the patients presenting with cardiac tamponade, 50% had acute pancreatitis on admission (RR = 1.25, 95% CI: 0.34 to 4.48, and *p* value = 0.73). Pericardiocentesis was done in a total of 11 patients (73.3%). Of the patients who underwent pericardiocentesis, eight patients recovered (72.7% recovery). Endoscopic retrograde cholangiopancreatography (ERCP) was used in 40% of the patients to diagnose PPF. Computed tomography (CT) abdomen was used to make the diagnosis in 26.6% of patients. Two patients had CT angiogram of the chest to rule out pulmonary embolism that revealed suspected pancreaticopericardial fistula, which was later confirmed by MRCP. Three cases were diagnosed by direct surgical exploration of the pancreatic duct. For definitive treatment, six patients underwent surgery (40%) with complete recovery in all six patients. Endoscopic retrograde cholangiopancreatography (ERCP) was done for four patients (26.6% cases) of which two patients recovered completely and two patients died. Two patients underwent pericardial window only (13.3%) of which only one patient recovered. One patient was managed conservatively without any invasive intervention. An external transhepatic drain was placed in one patient. Endoscopic ultrasound guided cystgastrostomy was done in one patient. All three patients had a complete recovery.

## 5. Discussion

A pancreatic fistula (PF) is defined as an abnormal connection between the pancreas and adjacent or distant organs, structures, and spaces [15]. PFs are classified as “internal” if the pancreatic duct communicates with the peritoneal or pleural cavity or any other hollow viscus and “external” if the pancreatic duct communicates with the skin [1]. The pathogenic mechanism of both internal and external PFs consists of disruption of the pancreatic duct secondary to pancreatitis or trauma [16, 17]. Persistent leakage of pancreatic secretions can result in development of internal pancreatic fistulas to neighboring hollow viscus (colon, duodenum, stomach, and esophagus), body cavities (peritoneal, pleural, and mediastinal), and vasculature (splenic vein, portal vein, and aorta). If the communication occurs anteriorly into the peritoneal cavity, it results in pancreatic ascites. A posterior communication may track into the pleural cavity or mediastinum [18]. A mediastinal pseudocyst occurs if the secretions are confined to mediastinum [18]. However, penetration of the secretion through pleura into pleural cavities, pericardium, or bronchial tree results in pancreaticopleural, pancreaticopericardial, or pancreaticobronchial fistula, respectively.

### 5.1. Incidence

The incidence of PFs after pancreatic resection ranges from 10 to 29% depending upon the type of surgery and underlying pancreatic pathology [15, 16]. The exact incidence of thoracopancreatic fistula and especially pancreaticopericardial fistula (PPF) is unknown. A review of English medical literature extending from 1965 to 1990 showed only 89 cases of thoracopancreatic fistulas [19]. The incidence of pancreaticopleural fistula has been reported in roughly 0.4% of patients with pancreatitis and 2.3–4.5% of patients presenting with pancreatic pseudocyst [20]. Our report indicates an increased incidence of pancreaticopericardial fistula in patients with existing history of chronic pancreatitis (73.3%). Acute pancreatitis was present in 42% of patients with PPF on admission. It is interesting to note that, of the patients with acute pancreatitis, 66% did not have any history of chronic pancreatitis. This association could be due to an undetected chronic subclinical pancreatitis in this patient population. However, we cannot exclude the possibility of acute pancreatitis resulting in fistula to pericardium based on the available information.

#### 5.1.1. Clinical Presentation

The clinical symptoms of thoracopancreatic fistula include cough, shortness of breath, chest pain, wheezing, palpitations, and dysphagia [21]. Patients with pancreaticopleural fistula can have unilateral and bilateral pleural effusions with dullness to percussion over the thorax and diminished breath sounds on physical examination. Our study indicates that patients with PPF are predominately male with median age of 43 years and a strong history of alcoholism (78%). Dyspnea is the most common clinical symptom (78%) followed by hypotension (53%), chest pain (50%), fever (43%), abdominal pain (26.6%), syncope (6.6%), weight loss (6.6%), and ascites (6.6%). Cardiac tamponade was present in 53% of patients, and of all the patients with cardiac tamponade 75% had history of chronic pancreatitis (RR = 0.74, 95% CI: 0.32 to 1.67, and *p* value = 0.46). Because of small sample size and the fact that PPF is a rare disease, the relative risk and confidence interval cannot be interpreted inaccurately. However, given that majority of patients with PPF had past medical history of chronic pancreatitis, a PPF should be suspected in patients with chronic pancreatitis presenting with cardiac tamponade.

#### 5.1.2. Diagnosis

The diagnosis of thoracopancreatic fistula should be suspected in patients with history of pancreatitis, pancreatic trauma, or surgery who present with a combination of abdominal and chest symptoms (dyspnea, orthopnea, chest pain, wheezing, etc.). The diagnosis is established by the finding of high levels of amylase in extravasated pancreatic fluid and evidence of duct disruption on imaging. For pancreaticopleural fistulas, the pleural effusions are exudative and amylase-rich with pleural fluid amylase greater than the upper limit of normal for serum amylase or a pleural fluid to serum amylase ratio greater than 1.0 [22]. To date there is no published data regarding the cardiac fluid analysis in patients with pancreaticopericardial fistula. Our study indicates that the cardiac fluid in such patients is exudative and amylase-rich in nature with pericardial fluid amylase to serum amylase ratio greater than 1.0. Our case and three more cases found an increased level of pericardial fluid lipase levels in patients with PPF.

### 5.2. Imaging

A number of imaging studies have been proposed to diagnose internal pancreatic fistulas. A CT scan of abdomen may demonstrate free fluid and walled-off collections in abdominal and thoracic cavities and changes of acute and chronic pancreatitis. However, a CT scan is diagnostic of pancreatic fistula only if performed immediately after an ERCP when it may demonstrate a fistulous tract [23]. MRCP is also another noninvasive method of diagnosing fistulas and should be performed if there is concern for adjacent complications (example is a pseudoaneurysm on CT) or if an ERCP is not performed. MRCP has advantage of guiding the clinical management by delineating the pancreatic duct injuries including those upstream of a complete duct disruption that would not be visualized on ERCP [24, 25]. ERCP provides direct evidence of a pancreatic fistula and is the test of choice if therapeutic pancreatic stenting is planned [26]. Unlike abdominal CT or MRCP, ERCP has the ability to demonstrate contrast filling the pancreatic ducts and extravasation in real time. ERCP has higher sensitivity and specificity for pancreatic duct leak as compared to CT scan [27]. Our report indicates that 40% of patients with pancreaticopericardial fistula were diagnosed by ERCP. CT abdomen was used to make the diagnosis in 26.6% of patients. Two patients had CT angiogram of the chest to rule out pulmonary embolism which revealed suspected pancreaticopericardial fistula which was later confirmed by MRCP. Three cases were diagnosed by direct surgical exploration of the pancreatic duct. In the above-mentioned patient the CT scan of chest on admission was done without contrast and failed to demonstrate the fistula. However CT angiogram of chest done in later hospital course demonstrated the fistula, indicating that contrast enhancement is required for visualization of fistulous tract.

### 5.3. Management

The management of pancreatic fistula is based on case reports and small observational studies [28]. The initial care depends on the presence of symptoms of abdominal pain, fever, and hypotension; presence of pancreatic necrosis on imaging and associated complications of infection; septic shock; and so forth. Reducing pancreatic stimulation by maintaining nil-by-mouth (NPO) status is part of initial management. Nasojejunal feeding to correct malnutrition is essential, and enteral nutrition is associated with lower incidence of infection, higher 30-day fistula closure rates, and shorter time to closure of postoperative pancreatic fistula as compared to total parenteral nutrition [29–31]. Octreotide 100 mcg three times daily in patients with high output fistula has been reported to be effective in reducing the fistula output, but it does not affect rate of fistula closure [29]. Fifty to sixty-five percent of internal fistulas close with supportive care [32]. In patients with persistent symptomatic fistula, additional interventions are required. Endoscopic treatment is preferred approach for management of most pancreatic fistulas. The goal of endoscopic therapy is internal drainage of pancreatic secretions, which thereby reduces flow through the fistula tract via placement of a pancreatic stent and/or pancreatic sphincterotomy [33, 34]. In a case series, endoscopic therapy for pancreatic fistulas has been associated with success rates of 85–100% [32]. Surgery for pancreatic fistula is indicated when endoscopic management fails or is technically difficult and is associated with a success rate of 90% with an associated mortality of 6–9% [32]. In case of pericardial tamponade, pericardiocentesis and pericardial drain placement becomes essential for initial stabilization of the patient followed by definitive treatment. All patients in our study who presented with acute tamponade had pericardial effusion drained. Four patients underwent ERCP guided stenting (26.6%) of which two patients recovered while two patients died. Six patients underwent surgical treatment (40%) with complete recovery in all six patients. Our patient underwent endoscopic ultrasound guided cystgastrostomy with complete recovery. Pericardial window was done as primary treatment in two patients (13.3%) of which one patient survived. Two patients were managed conservatively and one patient eventually had a transhepatic drain placed. Both these patients recovered without any complications.

## 6. Conclusion

PPF is a rare complication of pancreatitis. To our knowledge this is the first paper on this subject discussing the patient demographics, clinical picture, diagnostic modalities, and treatment options. The data is limited owing to rarity of the disease and no randomized trials are available that guide the diagnosis and treatment options. Identification and reporting of cases are required to have a better understanding of the disease process, which can affect treatment modes and patient outcomes.

## Figures and Tables

**Table 1:** Pancreaticopericardial fistula cases from 1971 to 2014 with patient demographics, symptoms, diagnostic test, and treatments.

Author	Year	Sex	Age	Alcohol	Chronic pancreatitis	Acute pancreatitis	Presenting symptoms	Dyspnea	Abdominal pain	Fever	Hypotension	Chest pain	Tamponade physiology	Pericardial drain	Diagnostic modality	Serum amylase	Pericardial amylase	Treatment	Patient outcomes
Lipson and Stephenson [1]	1971	M	40	N	Y	N	Ascites	N	N	N	N	N	N/A	N	Surgical exploration	613	N/A	Surgical	Recovered

Davidson et al. [2]	1979	M	39	Y	Y	N	Chest pain	Y	N	Y	N/A	Y	N	Pericardiocentesis	Surgical exploration	2000	12700	Surgical	Recovered

Imai et al. [3]	1993	M	42	Y	Y	Y	Dyspnea	Y	N	N	N	N	Y	Pericardiocentesis	ERCP	1681	6094	Surgical	Recovered

Schoonjans et al. [4]	1996	M	42	Y	Y	Y	Impending tamponade	Y	N	Y	N	N	N	N	ERCP	1413	N/A	Conservative management	Recovered

Tan et al. [5]	2002	M	28	Y	Y	N	Weight loss	Y	N	Y	N	N	Y	Pericardial drain	CT scan	N/A	5900	Surgical	Recovered

Oláh et al. [6]	2002	M	41	Y	Y	N	Dyspnea	Y	N	Y	Y	Y	Y	Pericardiocentesis	CT scan	748	226	Ultrasound guided drain placed transhepatically	Recovered

Balasubramanian et al. [7]	2004	M	16	N	Y	N	Dyspnea	Y					N	Pericardiocentesis	CT scan			Surgical	Recovered

François et al. [8]	2005	M	60	N/A	Pancreatic abscess	N/A	Back pain and dyspnea	Y	N	N/A	N/A	N/A	N/A	Pericardiocentesis	ERCP	20	328	Pericardial window	Recovered

Komtong et al. [9]	2006	M	43	Y	N	Y	Epigastric pain, dysphagia, orthopnea		Y	N	N	Y	N	N	ERCP and CT scan	285	N/A	ERCP stenting	Recovered

Ching et al. [10]	2007	M	45	Y	Y	N	Hemoptysis	Y	N	Y	Y	Y	N	N	CTA and MRCP	Normal	N/A	Surgical	Recovered

Bhatt et al. [11]	2011	M	47	Y	N	Y	Epigastric pain	Y	Y	N	Y	N	Y	Pericardial window		1830	N/A	Pericardial window	Death

Lamparter and Sundermann [12]	2013	M	57	Y	N	Y	Abdominal pain	N	Y	N	Y	N/A	Y	Y	ERCP	130	21560	ERCP stenting	Recovered

Parekh et al. [13]	2013	M	67	N	Y	Y	Chest pain	Y	N	N	Y	Y	Y	Pericardiocentesis	Surgical exploration	N/A	24000	ERCP stenting	Death

Sommer and Wilcox [14]	2014	M	58	Y	Y	N	Syncope	Y	N	N	N	N	Y	Y	ERCP	386	633	ERCP stenting	Death

Khan et al. (above reported case)	2014	M	45	Y	Y	N	Dyspnea	Y	N	N	y	Y	Y	Pericardiocentesis	CTA and MRCP	Normal	101	Endoscopic ultrasound & cystgastrostomy	Recovered

M = male, Y = yes, N = no, and N/A = not available.
